# Multimodal biomarker panel for early prediction of anastomotic leak after colorectal surgery: from inflammation to ischemia

**DOI:** 10.3389/fsurg.2026.1809885

**Published:** 2026-06-02

**Authors:** Yuji Li

**Affiliations:** Department of Gastrointestinal Surgery, The First Affiliated Hospital of China Medical University, Shenyang, Liaoning, China

**Keywords:** anastomotic leakage, biomarkers, colorectal surgery, C-reactive protein, gut microbiome, machine learning, peritoneal drain fluid, procalcitonin

## Abstract

Anastomotic leakage is one of the most serious complications following colorectal surgery, with an incidence ranging from 2% to 19%, and is closely associated with increased perioperative mortality, prolonged hospital stay, and poor oncological outcomes. Traditional clinical diagnosis relies on signs, symptoms, and imaging studies, which exhibit significant time delays. In recent years, researchers have explored early warning biomarkers from multiple perspectives including inflammatory response, tissue ischemia, microbial changes, and extracellular matrix remodeling, accumulating abundant research data. This article systematically reviews the current application status of serum inflammatory markers, peritoneal drain fluid cytokines, ischemic metabolites, microbiome markers, and tissue repair-related molecules in predicting anastomotic leakage, with emphasis on analyzing the diagnostic performance, optimal detection time windows, and clinical operability of various biomarker categories. Based on this foundation, we propose a multimodal prediction framework integrating four dimensions of “inflammation-ischemia-microbiome-tissue repair” and discuss the challenges in translating this framework into clinical decision-making tools. Machine learning algorithms demonstrate application potential in integrating multi-source heterogeneous data, but insufficient external validation remains the primary bottleneck constraining clinical implementation. Future research directions should focus on large-scale multicenter prospective cohort validation, establishment of standardized detection protocols, and development of implantable real-time monitoring technologies.

## 1 Introduction

Anastomotic leakage (AL) is the most challenging complication following colorectal surgery, with reported incidence rates ranging from 2% to 19%, and can exceed 20% in low rectal anastomoses (1, 2). The consequences of AL include reoperation, intra-abdominal infection, sepsis, prolonged hospitalization, and increased risk of perioperative mortality (3, 4). In addition, AL has been associated with adverse oncological outcomes and impaired postoperative quality of life after colorectal resection (5, 6).

Early diagnosis of AL remains challenging. Traditional evaluation relies on non-specific clinical findings together with imaging modalities such as abdominal CT, which are useful for confirming suspected leakage but remain limited as early predictive tools (7). This reactive diagnostic approach delays the optimal intervention window and increases the risk of adverse outcomes. Therefore, identifying biomarkers capable of warning of AL before clinical symptoms appear holds significant clinical value.

From a pathophysiological perspective, the occurrence of AL involves multiple interrelated pathological processes: inadequate blood supply leads to local ischemia, ischemia triggers anaerobic metabolism and tissue damage, damage activates inflammatory cascade reactions, and the inflammatory environment in turn affects tissue repair and bacterial colonization (8). These biological mechanisms are superimposed on patient- and procedure-related susceptibility factors, including low anastomosis, comorbidity burden, nutritional status, and operative complexity (9). This multifactorial pathological mechanism suggests that, theoretically, warning signals can be sought from multiple dimensions including ischemia, inflammation, microbiome, and tissue remodeling.

Existing research exhibits obvious fragmentation characteristics. Different research teams have separately focused on serum C-reactive protein (CRP) (10), peritoneal drain fluid cytokines (11), gut microbiome changes (12), and other different directions, lacking systematic integration. A systematic review published by Litchinko et al. (9) indicated that existing AL prediction scoring systems exhibit significant heterogeneity, are mostly based on small-sample single-center cohorts, and severely lack external validation.

This article aims to construct an integrative framework for a multimodal biomarker prediction system. Through systematic review of research evidence for various biomarker categories, analysis of their advantages and limitations, and discussion of how to integrate dispersed information into operable clinical tools, we provide directional references for future research in this field.

## 2 Methods of literature review

### 2.1 Literature search strategy

This review was conducted as a structured narrative review rather than a formal systematic review or meta-analysis. A targeted literature search was performed to improve transparency and to align the review with current expectations for high-quality narrative reviews. The principal databases consulted were PubMed, Web of Science, and Scopus. The search strategy focused on studies published from January 2000 to January 2026 and used combinations of the following keywords: “anastomotic leak”, “anastomotic leakage”, “colorectal surgery”, “rectal surgery”, “biomarker”, “C-reactive protein”, “procalcitonin”, “interleukin-6”, “drain fluid”, “peritoneal cytokine”, “matrix metalloproteinase”, “lactate”, “pH”, “intestinal fatty acid-binding protein”, “microbiome”, “indocyanine green”, and “machine learning”. Priority was given to systematic reviews, meta-analyses, randomized controlled trials, multicenter prospective studies, and representative translational investigations with clear clinical relevance. Additional publications were identified by screening the reference lists of eligible studies. Studies focusing on non-colorectal anastomoses or lacking adequate methodological detail were not prioritized. Because the literature is highly heterogeneous with respect to endpoints, timing, and assay methodology, a quantitative synthesis was not attempted. The interpretive aim of the present review is critical synthesis. Accordingly, each biomarker category is discussed in terms of biological rationale, timing of signal emergence, level of evidence, reproducibility, validation status, and practical accessibility in routine colorectal care.

### 2.2 Evidence hierarchy and interpretive approach

To avoid placing exploratory studies and high-level evidence on the same interpretive level, the evidence in this review was appraised according to study design and translational maturity. Meta-analyses, systematic reviews, and randomized controlled trials were regarded as providing the highest level of clinical evidence, although their findings still require interpretation in light of outcome heterogeneity. Prospective observational studies were considered intermediate-level evidence, particularly when sampling windows and endpoints were prespecified. Retrospective cohorts, pilot studies, and single-center exploratory analyses were interpreted more cautiously because of higher risks of selection bias, overfitting, and limited generalizability. Accordingly, the discussion throughout this review emphasizes not only reported diagnostic performance, but also reproducibility, external validation, practical accessibility, and readiness for routine clinical implementation. A structured overview of the evidence maturity, key limitations, and current clinical readiness of representative biomarker domains is provided in Table 1.

**Table 1:** Evidence maturity and clinical readiness of representative biomarker domains.

**Domain**	**Representative markers/tools**	**Typical signal window**	**Evidence maturity**	**Main strengths**	**Key limitations**	**Current readiness**
Systemic inflammation	CRP	POD3-5	High	Widely available; dynamic rule-out utility	Limited specificity	Routine
Systemic infection-oriented	PCT	POD3-5	Moderate	More infection-oriented than CRP	Higher cost; inconsistent incremental value	Selective adjunct
Composite blood indices	CAR, SII, NLR	POD1-3	Low-Moderate	Low cost; routine inputs	Variable formulas and thresholds	Exploratory adjunct
Peritoneal cytokines	IL-6, IL-10, TNF-α	POD1-3	Moderate	Lesion-proximal and earlier local signal	Require drains; poor standardization	Selected/high-risk settings
Matrix remodeling	MMP-8, MMP-9	Early postoperative	Low	Mechanistically linked to failed healing	Small cohorts; assay complexity	Research
Ischemia-related	Lactate, L/P ratio, pH, I-FABP	POD0-5	Low-Moderate	Capture upstream perfusion injury	Sampling constraints; limited validation	Research/selected settings
Intraoperative adjunct	ICG fluorescence angiography	Intraoperative	Moderate	Immediate perfusion assessment	Not a postoperative biomarker; RCT results mixed	Adjunctive
Microbiome/bacterial	Mucosal microbiome, E. faecalis PCR	Preop or POD3	Low	Biologically rich; may identify high-risk phenotypes	Highly exploratory; workflow variability	Research
Data integration	Machine learning models	Depends on inputs	Low-Moderate	Can integrate multidimensional data	External validation remains insufficient	Investigational

## 3 Pathophysiological basis of anastomotic healing

### 3.1 Normal healing process and failure mechanisms

Intestinal anastomotic healing proceeds through three overlapping phases: the inflammatory phase (postoperative days 0–4), the proliferative phase (postoperative days 3–14), and the remodeling phase (weeks to months postoperatively) (13). The inflammatory phase is characterized by neutrophil and macrophage infiltration, clearing necrotic tissue and releasing cytokines, laying the foundation for subsequent repair. The core events of the proliferative phase are fibroblast activation and migration, synthesizing collagen to form granulation tissue. The remodeling phase involves collagen cross-linking reorganization and restoration of tissue strength.

AL is essentially a failure of the healing process. Krarup et al. (14) classified risk factors into three categories: technical factors (poor blood supply, anastomotic tension), patient factors (malnutrition, diabetes, immunosuppression, preoperative chemoradiotherapy), and anatomical factors (low rectal anastomosis). Among these, local blood supply is the most critical determining factor. Under hypoxic conditions, fibroblast function is impaired, collagen synthesis is reduced, and anastomotic mechanical strength cannot be established (15). In addition, contemporary perioperative studies have shown that AL risk is also influenced by broader patient- and procedure-related factors, including age, male sex, obesity, nutritional status, operative duration, and intraoperative blood transfusion, which may interact with local healing failure and modify postoperative vulnerability (9, 16).

### 3.2 Special risks of low anastomosis

The AL risk of low rectal anastomosis is significantly higher than that of colonic anastomosis. A meta-analysis by Qu et al. (17) included 14 studies with 4,580 patients and found that patients with tumors ≤5 cm from the anal verge had significantly higher AL risk of those with tumors >5 cm from the anal verge (OR 9.63, 95% CI 3.05–30.43, *P* = 0.0001). This phenomenon involves multiple factors: low anastomosis is technically more difficult, the blood supply to the distal rectum is relatively weaker (dependent on branches of the middle and inferior rectal arteries), the tissue damage effects of preoperative neoadjuvant radiotherapy are more pronounced in the low position, and low anastomoses bear greater mechanical stress (18).

### 3.3 Impact of neoadjuvant therapy

Neoadjuvant chemoradiotherapy is the standard treatment regimen for locally advanced rectal cancer, but its adverse effects on anastomotic healing have been widely recognized. Radiotherapy causes vascular endothelial damage, tissue fibrosis, and microcirculatory disturbance, while chemotherapy inhibits cell proliferation and collagen synthesis (19, 20). This background factor needs to be considered when evaluating the predictive value of biomarkers.

## 4 Systemic inflammatory biomarkers

### 4.1 C-Reactive protein

CRP is currently the most clinically established biomarker for early AL surveillance. As an acute-phase reactant induced largely by IL-6, CRP follows a relatively predictable postoperative trajectory, typically rising on postoperative day 1, peaking around days 2–3, and subsequently declining in uncomplicated recovery. In patients who develop AL, this pattern often deviates, with persistently elevated values or secondary rises after the expected peak (21, 22).

Warschkow et al. (10) analyzed data from 1,832 colorectal surgery patients and established a CRP-based diagnostic model. CRP > 135 mg/L on postoperative day 4 predicted AL with a sensitivity of 68% and specificity of 83%. This study also emphasized that CRP dynamic changes have greater predictive value than single values. This concept is also supported by trajectory-based analyses, such as the PREDICT study, which suggested that postoperative CRP patterns may be more informative than isolated cutoff values for identifying patients at risk of AL (23).

The main limitation of CRP is insufficient specificity. Other postoperative complications such as pulmonary infection, wound infection, and venous thrombosis can all cause CRP elevation, resulting in false positives (24). Additionally, the optimal prediction time window for CRP is after postoperative day 3, by which time some patients have already developed clinical symptoms.

### 4.2 Procalcitonin

Research on procalcitonin (PCT) as an AL marker has received attention in recent years. PCT is a precursor peptide of calcitonin, with extremely low normal serum concentrations (<0.1 ng/mL), and is produced by neuroendocrine cells of various tissues during bacterial infection, causing serum concentrations to rise sharply (25). Compared with CRP, PCT has higher specificity for bacterial infection and responds weakly to simple tissue damage or viral infection.

A prospective observational study by Garcia-Granero et al. (26) included 205 colorectal resection patients and found that, for major anastomotic leaks, PCT and CRP were both reliable predictors on postoperative days 3–5 (AUC > 0.80, *p* < 0.0001). The best performance was achieved by PCT on postoperative day 5 (AUC = 0.86), with a cutoff of 0.31 ng/mL yielding 100% sensitivity, 72% specificity, and 100% negative predictive value. However, neither marker was reliable when minor AL was also included (maximum AUC < 0.80). This finding illustrates how differences in endpoint definition and leak severity classification may substantially influence the apparent diagnostic performance of postoperative biomarkers.

A multicenter prospective study by Facy et al. (27) involving 501 patients compared the diagnostic performance of CRP and PCT and found that CRP was significantly more discriminating than PCT on postoperative day 4 for intra-abdominal infection (AUC: 0.775 vs. 0.689, *p* = 0.03) and for all infectious complications (AUC: 0.783 vs. 0.671, *p* = 0.0002). The authors concluded that CRP should be systematically measured on postoperative day 4 as a more reliable tool.

A meta-analysis by Cousin et al. (28) pooled data from 11 studies with 2,692 patients and found that the diagnostic accuracy of CRP and PCT did not differ significantly at any postoperative day. On postoperative day 5, AUC was 0.87 for CRP and 0.90 for PCT. The authors concluded that PCT appeared to have no added value over CRP, though both markers below cutoff values between days 3 and 5 could help ensure safe early discharge.

The clinical implementation of PCT faces practical barriers including higher testing costs and being a non-routine laboratory test. Additionally, optimal threshold values in existing studies vary considerably, requiring further standardization.

### 4.3 White blood cell count and composite inflammatory indices

Compared with single conventional inflammatory indices such as WBC, recent studies have increasingly focused on composite blood-based markers, including neutrophil-to-lymphocyte ratio (NLR), platelet-to-lymphocyte ratio (PLR), systemic immune-inflammation index (SII = platelets × neutrophils/lymphocytes), and C-reactive protein-to-albumin ratio (CAR), to improve risk stratification using routine laboratory data (29). Among these indices, NLR has also been reported as a potentially useful inflammatory marker in recent colorectal AL studies, although the overall consistency of its predictive performance remains less robust than that of CRP (30).

A prospective cohort study by Goulart et al. (31) included 130 patients undergoing elective colorectal surgery and found that CRP on POD1 and CRP and CAR on POD3 were independent predictors of surgical site infection (AUC 0.639, 0.736, and 0.729, respectively), whereas WBC and PCT did not show significant predictive value. A prospective study by Qi et al. (32) evaluated serum and peritoneal biomarkers in 157 patients following laparoscopic low anterior resection and found that CAR on postoperative day 1 combined with SII and peritoneal IL-6 on postoperative day 3 were independent predictors of symptomatic AL, with the nomogram achieving a c-index of 0.812.

Composite indices remain attractive because they are inexpensive and based on routine laboratory inputs. However, variable formulas, thresholds, and endpoint definitions currently limit their comparability and generalizability, and further multicenter validation remains necessary.

### 4.4 Interleukin-6

IL-6 is an upstream cytokine in the inflammatory cascade and contributes to induction of acute-phase proteins such as CRP. Because IL-6 elevation may precede CRP, it is biologically attractive as an earlier warning signal for AL (33).

However, compared with CRP, the current clinical evidence for IL-6 remains less mature, and its routine application is limited by assay availability, cost, and lack of standardization across studies. Therefore, IL-6 is better regarded as a promising adjunctive marker than as a clinically established standalone tool.

### 4.5 Synthesis of systemic inflammatory biomarkers

Taken together, systemic inflammatory biomarkers remain the most clinically accessible domain for early AL surveillance, but the maturity of evidence differs substantially across markers. CRP currently has the strongest and most reproducible support, particularly as a dynamic marker measured on postoperative days 3–5 (34, 35). PCT is also a promising marker and may offer additional value in selected settings, although its incremental benefit over CRP has not been consistent across studies. Composite indices such as the CRP-to-albumin ratio and other inflammation-based composite scores are attractive because they rely on routine laboratory parameters, but current evidence remains limited by heterogeneous cutoff determination, endpoint definitions, and insufficient external validation. IL-6 and other upstream cytokines are biologically promising for earlier detection, but the supporting evidence is less mature and routine clinical implementation remains limited. Therefore, these markers should not be interpreted as being at the same level of evidentiary readiness or clinical applicability.

## 5 Peritoneal drain fluid biomarkers

### 5.1 Theoretical advantages and practical challenges of local markers

Peritoneal drain fluid originates directly from the surgical area and theoretically can reflect local pathological changes at the anastomotic site earlier and more sensitively than systemic markers. A systematic review by Su'a et al. (11) analyzed 36 studies and 51 biomarkers (including peritoneal drain fluid biomarkers) and concluded that predictive performance was not sufficiently ideal when used peritoneal drain fluid biomarkers alone, but accuracy improved when combined with systemic markers.

The clinical implementation of peritoneal drain fluid markers faces multiple obstacles. Enhanced Recovery After Surgery (ERAS) protocols generally do not support routine placement of peritoneal drains after colorectal anastomosis. However, systematic reviews, including Cochrane evidence, have not demonstrated clear reductions in AL with routine drainage, nor consistent increases in wound infection, reoperation, or hospital stay attributable to drainage itself (36–38). If an increasing number of patients do not have drains placed, monitoring strategies based on drain fluid will lose their foundation for application. Additionally, drain fluid composition is affected by factors such as drain tube position, drainage volume, and dilution effects, making standardization difficult.

### 5.2 Peritoneal cytokines

Peritoneal cytokines, particularly IL-6, have been summarized in review-level literature as among the most promising local inflammatory markers for early AL detection, although heterogeneity in sampling protocols and assay methods remains substantial (39, 40). Peritoneal drain fluid IL-6 has been most thoroughly studied. A prospective study by Matthiessen et al. (41) in 23 patients after anterior resection found that peritoneal IL-6 and IL-10 were significantly higher on POD 1 and 2 in patients who developed AL (*P* = 0.002 and *P* = 0.012 for IL-6, respectively), and peritoneal TNF-α was also significantly elevated on POD 1 (*P* = 0.011), all preceding clinical diagnosis (median day 6). A pilot study by Cuff et al. (42) in 42 patients further demonstrated that a metric combining peritoneal IL-6 and CRP levels could accurately diagnose AL at 48 h postoperatively using automated immunoassay platforms already available in hospital laboratories.

Research on peritoneal TNF-α across different populations has yielded variable results. Yamamoto et al. (43) found that intraperitoneal TNF-α levels correlated with both intraperitoneal sepsis at laparotomy and postoperative septic complications in IBD patients, but showed no correlation with systemic inflammatory markers such as plasma cytokines and CRP, suggesting peritoneal and systemic inflammatory responses may be dissociated. This highlights the potential added value of peritoneal over systemic biomarker monitoring, though standardization of collection methods and assay platforms remains a key challenge.

### 5.3 Matrix metalloproteinases

Matrix metalloproteinases (MMPs) are zinc-dependent endopeptidases that degrade extracellular matrix and play key roles in tissue remodeling. Excessive MMP activity destroys newly formed collagen, leading to healing failure (44).

Pasternak et al. (45) analyzed peritoneal drain fluid MMP levels in 29 patients after low anterior resection and found that patients who developed AL had significantly elevated MMP-9 (median difference 29%, *P* = 0.03) and MMP-8 (median difference 58%, *P* = 0.02) in early postoperative intraperitoneal fluid, while no differences were observed for other MMPs or TIMPs. A review by Gray et al. (46) further highlighted MMPs among promising candidate biomarkers alongside ischaemic metabolites, inflammatory markers, and bacteria, though no AL biomarker has yet been validated in large-scale clinical trials.

A study by Shogan et al. (47) revealed a mechanistic link between MMP-9 and intestinal bacteria: Enterococcus faecalis, through its gelE and sprE genes, can degrade collagen and activate host tissue MMP-9, contributing to anastomotic tissue breakdown. Notably, topical antibiotics or pharmacological suppression of MMP-9 prevented AL in rat models, whereas standard intravenous antibiotics failed to eliminate E. faecalis at anastomotic tissues. This finding bridges tissue repair markers with microbial factors and suggests potential therapeutic targets.

## 6 Ischemia-related biomarkers

### 6.1 Lactate and anaerobic metabolism

Local ischemia is a core pathological mechanism of AL, and markers reflecting anaerobic metabolism possess theoretical predictive value. Lactate, the end product of anaerobic glycolysis, accumulates abundantly during tissue hypoxia. A systematic review by Wright et al. (48) analyzed the diagnostic value of peritoneal drain fluid biomarkers and found that lactate was “consistently shown to be elevated in patients with intra-abdominal complications and ALs,” while pH demonstrated excellent specificity. Daams et al. (49) used microdialysis to measure peritoneal metabolites in 24 patients following colorectal surgery and found that the mean lactate/pyruvate (L/P) ratio in AL patients was 25 (SD 4.7) compared with 19.2 (SD 3) in those without AL, and lactate levels were significantly higher during the first five postoperative days. The L/P ratio is considered superior to lactate alone in reflecting tissue oxygenation, as it controls for systemic metabolic variables. Matthiessen et al. (41) monitored metabolic changes using microdialysis catheters placed near the anastomosis and found that the intraperitoneal L/P ratio was significantly elevated on postoperative days 5 and 6 in patients who subsequently developed clinical AL, preceding clinical diagnosis. Ellebæk Pedersen et al. (50) similarly demonstrated in 45 low anterior resection patients that elevated L/P ratios occurred several days before the onset of clinical symptoms in patients with late AL, confirming the early warning potential of this indicator.

### 6.2 Ph monitoring

Peritoneal fluid pH is a straightforward ischemia indicator. Tissue ischemia triggers lactate accumulation and a local decrease in pH. A study by Millan et al. (51) in 90 rectal or sigmoid cancer patients using tonometry found that an anastomotic intramucosal pH <7.28 within the first 24 postoperative hours was associated with a 22-fold increased AL risk (*P* = 0.001 on multivariate analysis). Yang et al. (52) retrospectively studied 753 patients and confirmed that a persistently declining drain pH reached diagnostic significance from postoperative day 3 onward. pH monitoring offers the advantages of simplicity, rapidity, and low cost, and can be performed at the bedside. However, its sensitivity varies across studies, rendering it more useful as part of a combined assessment than as a standalone exclusionary test.

### 6.3 Intestinal fatty acid-binding protein

Intestinal fatty acid-binding protein (I-FABP) is a cytoplasmic protein of small intestinal enterocytes that is released into the circulation during intestinal ischemia or injury. Compared with lactate, I-FABP exhibits higher intestinal specificity and is unaffected by systemic metabolic status. Schellekens et al. (53) demonstrated in a human translational model that plasma I-FABP levels correlated closely with the morphologic severity of epithelial intestinal damage (*r* = −0.82, *P* < 0.001). Derikx et al. (54) conducted a systematic review confirming I-FABP as a reliable serological marker for human intestinal ischemia. However, research on I-FABP specifically for AL prediction after elective colorectal surgery remains in its preliminary stages and lacks large-scale validation.

## 7 Microbiome and bacterial markers

### 7.1 Gut microbiome and anastomotic healing

The role of bacteria in the pathogenesis of AL was recognized as early as the 1950s, and research in the microbiome era has provided new depth. Alverdy et al. (8) proposed the “pathogen virulence hypothesis,” positing that perioperative stress transforms commensal bacteria into pathogenic phenotypes that directly disrupt anastomotic healing. van Praagh et al. (12) analyzed the anastomotic mucosal microbiome of 123 colorectal surgery patients and reported that those who subsequently developed AL exhibited significantly lower microbial diversity and a higher relative abundance of Bacteroidaceae and Lachnospiraceae families. Lehr et al. (55) explored the potential of the preoperative mucosal microbiome as a predictive tool and found that microbial composition prior to surgery could differentiate patients who would and would not subsequently develop AL, offering new avenues for preoperative risk assessment. However, these microbiome-based findings remain exploratory and are currently constrained by limited cohort sizes, methodological heterogeneity, and the absence of standardized protocols for routine clinical application.

### 7.2 Bacterial PCR detection

A more direct bacterial detection strategy involves analyzing microorganisms in peritoneal drain fluid. Komen et al. (56) initially demonstrated that RT-PCR could detect colonic flora including *Escherichia coli* and *Enterococcus faecalis* in drain fluid. In the subsequent multicenter APPEAL study, Komen et al. (57) used PCR detection of *E. faecalis* DNA as a screening tool and found that patients with symptomatic AL had significantly increased *E. faecalis* concentrations on postoperative day 3 (diagnostic OR 31.6, *P* < 0.003), providing warning 1–2 days before clinical symptoms appeared. *Enterococcus faecalis* has attracted particular attention because of its collagenase activity. Shogan et al. (47) confirmed in an animal model that *E. faecalis* degrades collagen and activates host MMP9, thereby directly disrupting anastomotic tissue healing.

### 7.3 Impact of perioperative antibiotics

Perioperative antibiotics are standard measures for preventing surgical site infections; however, they inevitably alter gut microbiome composition. Guyton and Alverdy (58) reviewed the interrelationship between gut microbiota and gastrointestinal surgery, noting that antibiotic exposure leads to decreased microbial diversity and selective expansion of opportunistic pathogens such as Enterococcus. These alterations may be associated with increased AL risk. Antibiotic type, timing, and duration may all influence the microbiome and subsequent healing outcomes.

## 8 Intraoperative perfusion assessment technologies

### 8.1 Indocyanine green fluorescence angiography

Although intraoperative perfusion assessment is not a biomarker in the strict biochemical sense, it is included here because ischemia is a major upstream mechanism in anastomotic leak pathogenesis and perfusion-related information may complement postoperative biomarker-based surveillance. Indocyanine green (ICG) fluorescence angiography is the most extensively studied intraoperative perfusion assessment technology in recent years. After intravenous injection, ICG binds to plasma proteins and emits fluorescence under near-infrared excitation, enabling real-time visualization of tissue perfusion (59). The rationale for ICG use in colorectal surgery is to assess the blood supply of the intestinal segment prior to anastomosis construction and, if perfusion is inadequate, to extend the resection to a better-perfused area. Retrospective studies and meta-analyses of observational data have shown that ICG fluorescence angiography can reduce AL incidence (60).

However, results from individual RCTs have been inconsistent. De Nardi et al. (61) conducted a multicenter RCT enrolling 240 patients undergoing laparoscopic colorectal resection and found no statistically significant difference in AL rates between the ICG and control groups (5% vs. 9%, *P* > 0.05). The AVOID trial by Faber et al. (62) enrolled 907 patients undergoing minimally invasive left-sided colorectal surgery and reported no significant difference in AL rates (5.3% vs. 7.1%). More recent randomized evidence has remained mixed, with the IntAct trial similarly highlighting that the clinical effect of ICG-guided perfusion assessment may depend on operative context, implementation, and interpretation rather than on fluorescence imaging alone (63). Interpretation of these results involves multiple considerations: ICG assessment relies heavily on subjective surgeon judgment and lacks objective quantification standards; normal intraoperative perfusion cannot exclude ischemia arising from postoperative hemodynamic changes; and AL is a multifactorial process in which blood supply represents only one contributing factor. In addition, ICG should be interpreted within the broader context of intraoperative risk-reduction strategies, because other techniques such as intraoperative endoscopic testing may also help identify technical defects or bleeding points that are not captured by perfusion assessment alone (64).

### 8.2 Quantified perfusion parameters

To overcome the limitations of subjective assessment, researchers have explored objective quantification of ICG fluorescence parameters, including time to fluorescence (T0), fluorescence rise slope, and maximum fluorescence intensity (65). Son et al. (66) performed quantitative analysis of colon perfusion patterns using ICG angiography in laparoscopic colorectal surgery and confirmed that quantified parameters may have greater predictive value than subjective assessment, though standardization remains a key challenge.

## 9 Machine learning prediction models

### 9.1 Technical tools for multi-parameter integration

Machine learning (ML) is discussed here not as a biomarker itself, but as an integrative analytic approach that may combine heterogeneous biomarker and clinical data within future multimodal prediction systems. ML has advantages in integrating multi-source heterogeneous data and has been widely applied to AL prediction model development. Commonly used algorithms include logistic regression, random forest, gradient boosting (XGBoost), and neural networks. Taha-Mehlitz et al. (67) used multiple ML algorithms including random forest to construct a prediction model based on 21 preoperative factors from 1244 patients across four Swiss hospitals. The Random Forest model achieved an AUC-ROC of 0.78 on the internal test set but decreased significantly to 0.60 on the external holdout set. This pattern of “good internal performance, failed external validation” highlights issues of data heterogeneity and overfitting.

### 9.2 Dynamic analysis of CRP trajectories

Utilizing postoperative multi-timepoint biomarker trajectory data, rather than single static values, may provide richer predictive information. Woffenden et al. (68) compared ML models using CRP dynamic data and found that ML approaches demonstrated superior discrimination compared to conventional univariate CRP thresholds, balancing sensitivity and specificity more effectively.

### 9.3 Model integration strategies

Ochs et al. (69) adopted a meta-model ensemble strategy, integrating logits from multiple existing prediction models along with patient-specific clinical features across 13 European and North American centers (9,120 patients), achieving an F1 score of 87% on cross-validation and 70% on external validation. While ensemble approaches can theoretically combine the advantages of different models, the main limitation of ML models remains their “black box” nature, with insufficient clinical interpretability. Additionally, representativeness and quality of training data directly affect generalization ability. At present, most machine learning models in this field should be regarded as exploratory decision-support tools rather than clinically ready prediction systems, particularly in view of limited external validation and the risk of overfitting in small or heterogeneous datasets.

## 10 Emerging and translational directions

### 10.1 Implantable biosensors

Wahl et al. (70) reported a biodegradable implantable sensor that monitors anastomotic ischemia in real-time based on bioimpedance principles. This resorbable sensor can be integrated into circular staplers and successfully detected ischemia-related impedance changes in a porcine model. Continuous real-time monitoring without relying on intermittent sampling represents an important direction in monitoring technology development. The journey from proof-of-concept to clinical application remains long, with issues of biocompatibility, signal stability, degradation control, regulatory approval, and cost-effectiveness assessment still to be resolved. Accordingly, implantable biosensors should currently be viewed as promising proof-of-concept technologies rather than near-term tools for routine postoperative monitoring.

### 10.2 Point-of-care rapid testing technologies

Shortening the time from sampling to results is critically important for clinical decision-making. Point-of-care testing (POCT) devices for CRP have been commercialized and can yield results within 15 min. POCT technologies for PCT, IL-6, and other markers are under development (46). Multi-marker combined POCT platforms represent a future development direction.

### 10.3 Multimodal integration framework

The multimodal concept proposed in this review should be understood as a staged and pragmatic framework rather than a highly complex diagnostic algorithm. In current practice, the most realistic strategy is to combine baseline clinical risk stratification, intraoperative perfusion assessment where available, and postoperative dynamic monitoring centered on clinically accessible inflammatory markers. CRP remains the most feasible core marker for routine surveillance, whereas PCT may serve as an adjunct in selected settings. In patients with abdominal drains, local biomarkers such as peritoneal IL-6 or lactate may provide earlier localized warning, although their application depends on institutional practice and assay accessibility. More advanced approaches, including microbiome profiling, machine learning integration, and implantable sensing technologies, should currently be regarded as promising research directions rather than routine clinical tools. In practical terms, multimodal integration is intended to support earlier suspicion, more selective imaging, intensified surveillance in high-risk situations, and safer discharge decision-making, rather than to replace clinical judgment. A practical summary of this staged perioperative framework and its potential implications for management is shown in Table 2.

**Table 2:** Practical perioperative integration of multimodal surveillance for anastomotic leakage.

**Phase**	**Feasible tools**	**Purpose**	**How abnormal findings may influence management**
Preoperative	Clinical risk factors ± research microbiome	Estimate baseline leak vulnerability	Heighten vigilance; contextualize postoperative thresholds
Intraoperative	ICG fluorescence angiography where available	Assess gross perfusion adequacy	Consider revision of transection line or protective strategy
POD1-2	Clinical review; selective drain markers in drained high-risk patients	Detect early deviation from expected recovery	Increase bedside reassessment; delay premature discharge planning
POD3-5	Serial CRP ± PCT; optional local biomarkers if drains present	Core surveillance window for early leak suspicion	Lower threshold for CT imaging, maintain observation, escalate care
Post-discharge or extended surveillance	Symptom review, repeat labs/imaging in selected patients	Capture delayed or evolving leaks under ERAS	Prompt urgent reassessment or readmission if recovery deviates

## 11 Challenges and limitations

### 11.1 Heterogeneity in definitions and diagnostic criteria

The lack of unified standards for AL definition creates fundamental challenges for research. The grading system proposed by the International Study Group of Rectal Cancer (ISREC) (71) is widely cited, but in practice, whether to include asymptomatic radiological leaks and how to classify Grade A leaks requiring only conservative management varies across studies. Definition heterogeneity directly impairs comparability of research results. In addition, biomarker performance may differ substantially depending on whether studies focus on clinically significant symptomatic AL, radiological leaks detected through surveillance imaging, or composite endpoints that combine leaks of different severity. The timing of endpoint ascertainment also matters, because biomarkers evaluated for very early postoperative suspicion may perform differently from those assessed against leaks diagnosed later in the postoperative course. Accordingly, apparent differences in cutoff values or diagnostic accuracy across studies may partly reflect differences in endpoint definition rather than true biological inconsistency.

### 11.2 Sample size and statistical power

The relatively low incidence of AL (approximately 5%–10%) necessitates large-sample studies to obtain adequate statistical power. Most existing studies are small-sample, single-center investigations, often with only twenty to thirty events, leading to imprecise effect estimates and limited reliability of multivariable analyses. In addition, many currently available studies are retrospective and single-center in design, with limited external validation and an increased risk of selection bias. These methodological limitations should be considered when interpreting apparently favorable diagnostic performance reported in exploratory cohorts.

### 11.3 Clinical accessibility and cost-effectiveness

Ideal biomarkers should meet practical standards: simple testing, rapid results, acceptable cost, and clinical laboratory accessibility. By these criteria, CRP is almost the only fully qualified candidate. PCT testing is more costly and not routinely available, and cytokine and microbiome testing even more so. When translating research to clinical practice, practicality is a factor that must be considered (3).

### 11.4 Challenges in the enhanced recovery after surgery Era

The widespread implementation of ERAS protocols has transformed the landscape of postoperative monitoring. Early discharge (postoperative days 2–4) shortens the in-hospital observation window, and some AL may only manifest after discharge (36). Coping strategies include optimizing pre-discharge risk stratification, developing outpatient or home-based simple testing, and establishing post-discharge remote monitoring mechanisms.

## 12 Conclusions and future perspectives

Early prediction of AL is a multidimensional problem, and a single biomarker is unlikely to provide a complete solution. The evidence reviewed herein indicates that CRP is currently the most well-evidenced and clinically accessible inflammatory marker, with postoperative day 3–5 dynamic changes offering greater predictive value than single values; PCT may provide earlier warning signals but has inferior levels of evidence and clinical availability compared with CRP; peritoneal drain fluid markers are theoretically closer to the lesion site but are limited by standardization difficulties and restrictions on drain use under ERAS protocols; ischemia markers and microbiome markers remain at the research stage, some distance from clinical application; and RCT results for ICG fluorescence angiography did not consistently demonstrate benefit, suggesting that intraoperative assessment cannot entirely replace postoperative monitoring.

Based on the above evidence, the multimodal prediction framework should follow the principles of temporal integration, linking preoperative risk stratification, intraoperative assessment, and postoperative dynamic monitoring into a complete chain; multidimensional information fusion, comprehensively integrating signals from inflammation, ischemia, microbiome, and tissue repair; prioritizing practicality, balancing clinical operability while pursuing diagnostic accuracy; and individualized thresholds, adjusting alert levels according to baseline risk factors (low anastomosis, neoadjuvant therapy, etc.). In practical terms, the near-term value of multimodal integration lies in staged perioperative surveillance, more selective imaging, and safer discharge decision-making, rather than in the creation of a single standalone diagnostic algorithm.

Future research directions should include establishment of large-scale multicenter prospective cohorts providing sufficient event numbers and external validation; standardization of testing methods and analytical protocols enabling cross-study comparison; development of POCT technologies to shorten the time from sampling to results; clinical translation of real-time monitoring technologies such as implantable sensors; and rigorous validation of ML models to avoid false optimism caused by overfitting. Standardization of AL definitions, severity grading, and diagnostic time windows will also be essential for future validation studies, because heterogeneity in these endpoints directly affects the reported performance and comparability of candidate biomarkers. Through systematic integration of existing knowledge, identification of key gaps, and targeted filling of those gaps, this field is expected to make substantial progress in the coming years, ultimately improving patient outcomes and optimizing healthcare resource allocation.
